# A predictive model for prudent albumin priming in cardiopulmonary bypass circuits

**DOI:** 10.1051/ject/2026016

**Published:** 2026-09-15

**Authors:** Julie Fenske, Isabel Centner

**Affiliations:** University of Southern California Los Angeles CA USA

**Keywords:** Cardiopulmonary bypass, Albumin, Edema, Oncotic pressure, Colloid osmotic pressure, Fluid shift

## Abstract

*Background*: Dosing albumin to normalize colloid osmotic pressure is an underutilized strategy within the application of cardiopulmonary bypass. The negative sequelae of third-spacing and edema post-bypass are well known and documented within the literature, yet it is not common practice for perfusionists to calculate albumin dilution or tailor albumin administration to each individual patient. *Methods*: The author explored relevant literature to justify quantifying albumin dosing and concentration within the application of cardiopulmonary bypass. Sequelae following edema in the cardiac patient were also explored and presented, justifying the investigation of this topic. Formulas to quantify albumin dilution, resultant drop in colloid osmotic pressure, and suggested albumin dosing, were presented and unified into a model. *Results*: A spreadsheet-based model was developed to quantify albumin dilution that may be accessed or recreated by the reader for information purposes or for clinical use. This model enables the user to solely quantify their albumin dilution during bypass, or more proactively calculate albumin concentration and dosing in an attempt to mitigate large colloid osmotic pressure changes during bypass. *Conclusions*: Intentional albumin management can yield more physiologically normal albumin concentrations and colloid osmotic pressures undergoing cardiopulmonary bypass, reducing the sequelae previously described. The model presented enables informed albumin dosing by the perfusionist, and use of this model may be of especially great benefit to patients who are hypovolemic, underweight, or pediatric.

## Introduction

In cardiac surgery, cardiopulmonary bypass (CPB) enables a patient’s cardiopulmonary function to be temporarily replaced with an extracorporeal circuit (ECC). This mechanical support consists of a blood pump and a microporous membrane oxygenator, enabling the surgeon to arrest and operate on the heart without cessation of blood flow to the body. Prior to the initiation of CPB, the ECC is primed with a crystalloid solution and various additives that will intermix with the patient’s native circulating blood volume in order to establish a safe and air bubble-free circulation, aiming to minimize the physiologic disturbance inherently created by CPB. While protocols vary between institutions, the CPB prime usually consists of approximately 1200 mL crystalloid fluid and an added buffering agent, diuretic, anticoagulant, antifibrinolytic, antibiotic, and colloid albumin, which will be discussed in depth below.

**Figure 1:**
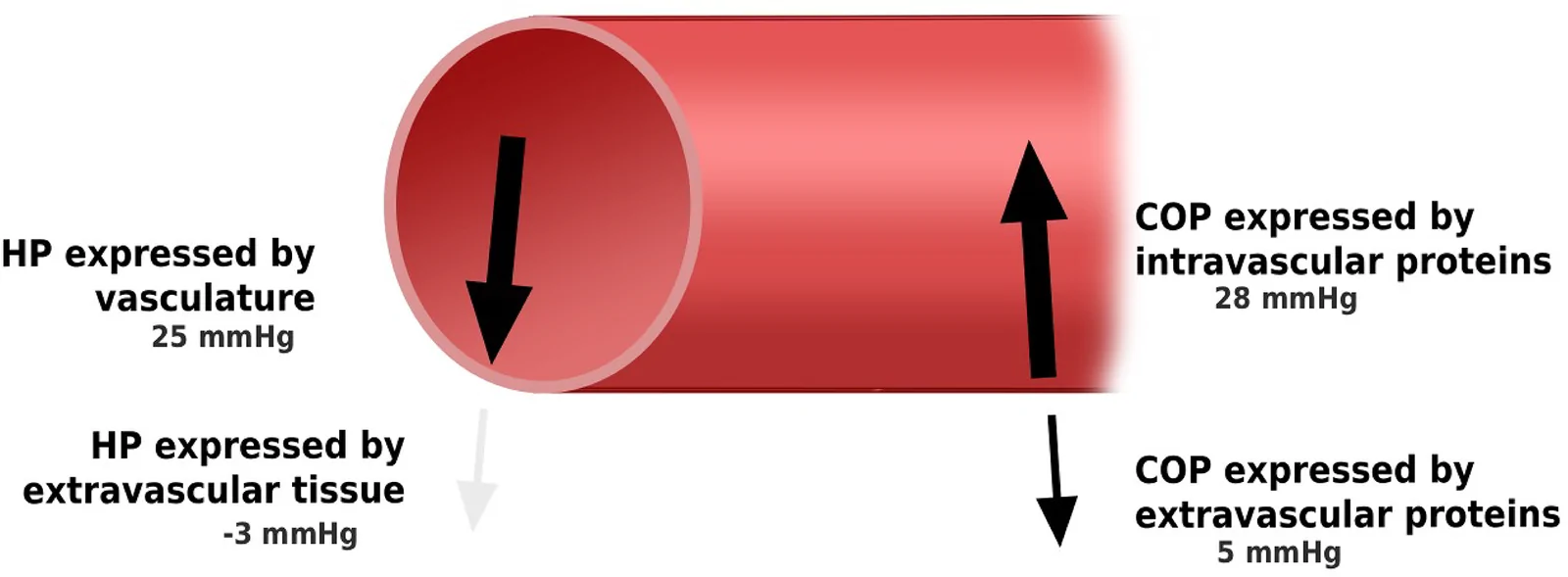
The Starling Forces are made up of the forces that motivate movement of fluid across the vascular wall. Figure created by the author.

Albumin, a natively occurring plasma protein, has a normal range of 3.4 g/dL to 5.4 g/dL in the human body; concentrations below or above this range are termed hypoalbuminemia and hyperalbuminemia, respectively. With an average molecular weight of 69,000 Daltons, albumin is impermeable to the capillary endothelium and is thus responsible for pulling water across the endothelium in an attempt to equalize colloid concentration intracapillary and extracapillary, creating an oncotic pressure, which will be referred to as colloid osmotic pressure (COP). Of the typical 28 mmHg of intracapillary COP, a majority (21.8 mmHg) is driven by albumin, with the remainder driven by other plasma proteins (globulins and, minimally fibrinogen). While net COP motivates water into the vasculature, hydrostatic pressure (HP) of a vessel motivates the movement of water out, as well as a small negative (at times, zero) pressure in the interstitium from the lymphatics and tissue matrix tension additionally pulling water into the tissue. Together these forces are described as the Starling Forces and generally balance with no dramatic pressure changes or fluid shift. Figure 1 illustrates the typical value of each Starling Force at the capillary level [1].

The HP of a capillary varies across the vessel; it is higher on the arterial end, and lowers as blood perfuses through to the venous end of the capillary due to vessel resistance and friction. With an arterial HP of ~35 mmHg and a venous HP of ~15 mmHg, the typical capillary HP is estimated to be in the mid-twenties (mmHg) [2,3]. The myogenic response of arterioles and precapillary sphincters allows the hydrostatic pressure of capillaries to remain consistent, even through systemic pressure changes seen during a CPB run.

A condition of acute hypoalbuminemia would reduce COP and disrupt the balance of the Starling Forces, prompting a net movement of water out of the vasculature [4]. When there is an excess of interstitial fluid, the pressure-responsive lymphatics work to drain fluid with a structure of smooth muscles that contract (lymphangions) and multiple one-way valves to return interstitial fluid (now called lymph) to lymph nodes and ultimately drain into the venous circulation. However, maximal stretch and dilation of lymph filaments, and therefore potential to drain excess fluid, is thought to plateau before interstitial pressure reaches +2 mmHg; lymph overwhelm occurs and edema results thereafter [4, 5]. Lymph drainage is further impaired by hypothermia and nonpulsatile flow, which are both common practices in CPB [6]. Mechanical positive-pressure ventilation and elevated CVP also impair the maximal capacity for lymph drainage, and excessive lymph drainage returning to the circulation easily perpetuates the cycle of fluid overload and resultant tissue edema [7].

In the patient undergoing CPB, edema is further exacerbated by a multitude of physiologic consequences. Capillary leak occurs following endothelial glycocalyx injury and damage by reactive oxygen species created by CPB, as well as the systemic inflammatory response inherent from contact with the artificial surfaces of the ECC [8, 9]. Further, conditions surrounding CPB (surgical stress, blood pressure changes, and temperature variation) all increase the concentration of circulating anti-diuretic hormone (ADH). Increased ADH initiates vasoconstriction, furthers capillary leak, and prompts uptake of free water in the collecting duct of the kidneys; all factors which continue to dilute blood albumin and increase hydrostatic pressure, encouraging further edema.

The multiple mechanisms creating tissue edema during CPB are certain and one can safely assume that edema will be a common, if not unavoidable, sequelae of CPB. In post-CPB patients, edema is often estimated as weight gain and the phenomenon of increased weight and edema following bypass has been documented and will be described below. Postoperative weight gain and edema are found to be associated with acute kidney injury (AKI), prolonged length-of-stay, pulmonary complications, blood transfusions, and overall mortality [10–12]. Resultant AKI and impaired glomerular filtration further perpetuate fluid retention and overload. In 2023, Koskinen et al. found that 59% of adult patients within their institution (*n* = 939) demonstrated ≥5% weight gain, which correlated with adverse outcomes post cardiac surgery [13]. A prospective 2011 study by Morin et al. (*n* = 109) found that, following coronary artery bypass surgery, weight gain increased the risk of major complications significantly when ≥5 kg weight gain versus 1–5 kg [14]. While hypoalbuminemia albumin is not the only factor influencing postoperative weight gain, maintenance of COP with albumin levels can mitigate the contribution of weight gain due to fluid shifting after albumin dilution.

## Methods

**Figure 2:**
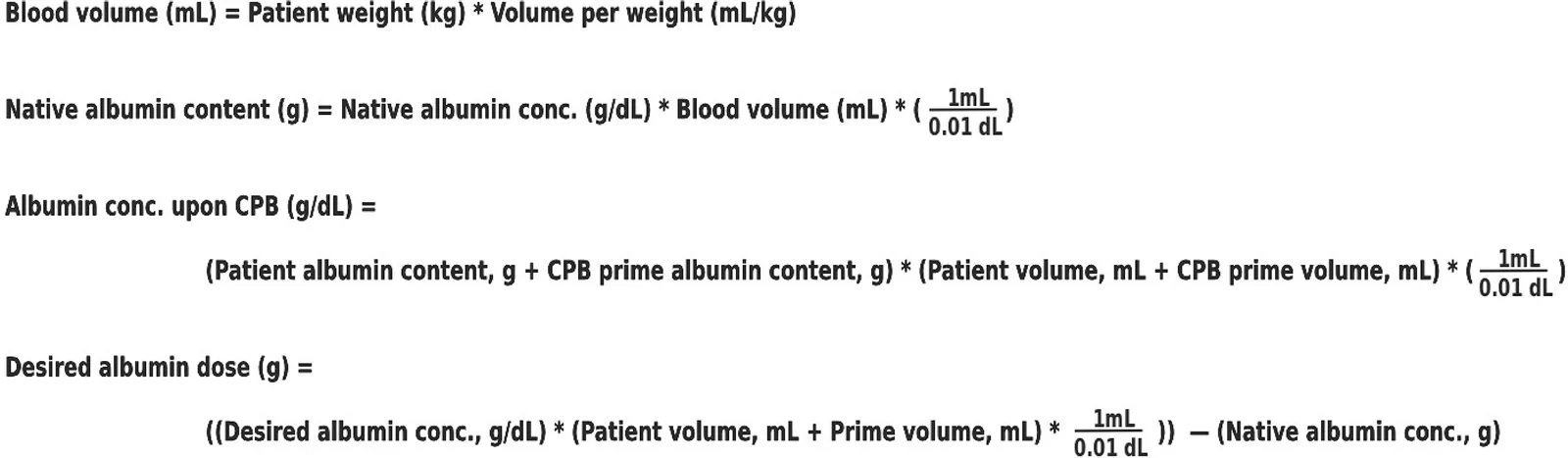
The variables and defining formulae identified prior to creating the predictive model.

The author first performed a literature search to gather and present information surrounding albumin and COP, fluid shift in the CPB and post-CPB patient, and sequelae of resultant edema; this data is presented in the introduction section of this paper. Based upon the equation set used to calculate dilutional hematocrit, the author established the following formulae to find albumin content within a patient, albumin concentration upon initiating CPB, and the dose of albumin to give to achieve a specified circulating concentration (Figure 2):

Following, the author used the free online spreadsheet software Google Sheets to create an automated model presented as a calculator-type interface. This software is analogous to Microsoft Excel and this model may be mirrored in any spreadsheet software able to compute simple formulae. The author’s version of the calculator may also be accessed with the URL presented in the results section below.

The calculator was created to be both straightforward and comprehensive, while ensuring safe and clear utilization by the user. First, any of the cells that do not request a value from the user are “locked”, keeping the descriptors and equations protected from incidental over-write. Second, any quantifiable cells were coded with an acceptable range (using the “data validation” tool, if criteria “is between”) presenting an error message if the user inputs a value out of a normal range, such as an albumin concentration of “45 g/dL” instead of “4.5 g/dL”. This will highlight and reject the input preventing inadvertent or incorrect calculations. Next, a “clear all” button is installed at the top of the spreadsheet to refresh the spreadsheet input field. This “clear all” function is also set to run any time the sheet is opened, and as well after 15 minutes of inactivity in order to prevent the carryover of prior inputs. Safety and intentionality are paramount and this model can only be used safely when requiring current and safe inputs from the user.

For comprehensive transparency, the formulae described above are also copied within the calculator, and the author’s contact information is listed, should a user have any feedback or concerns with use of the spreadsheet. Implementation of the predictive model is at the discretion of the user, who is expected to be knowledgeable about the formulae above and the contextual implementation of the model.

## Results

**Figure 3:**
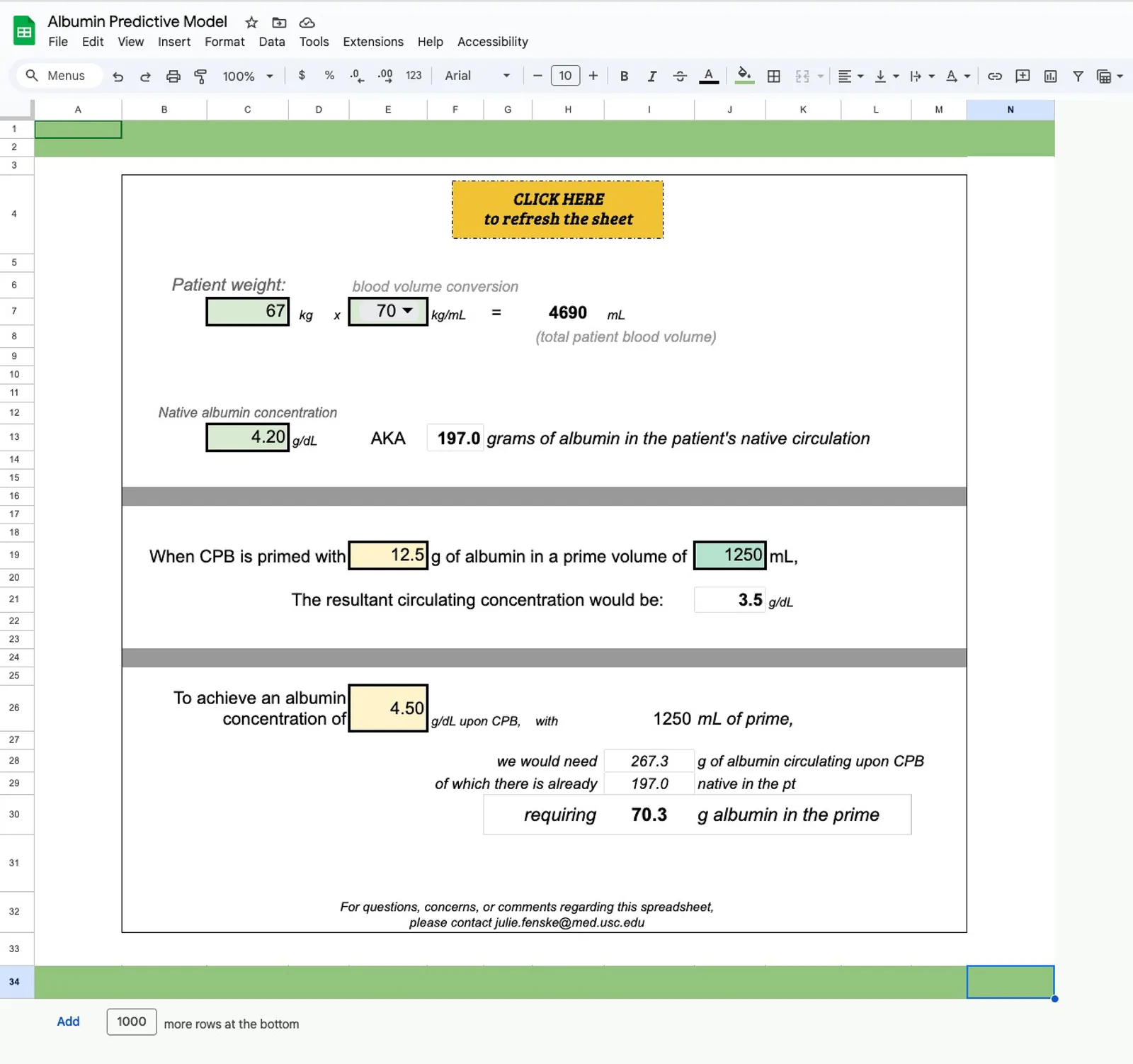
The interface of the predictive model while in use.

The author’s version of the calculator may be accessed at albuminmodel.short.gy/. The interface of the predictive model for albumin priming is shown in Figure 3.

## Discussion

A reduction in plasma albumin concentration will reduce the plasma COP, disrupting the Starling Forces and thereby the fluid balance between vasculature and the surrounding tissue, however it would be beneficial to quantify this action. In 1963, Landis et al set the precedent for quantifying the relationship between total plasma protein (TP) and COP, with COP = 2.1 TP + 0.16 TP^2^ + 0.009 TP^3^ [15]. From this we can extrapolate the effect of every 1 g/dL drop in albumin concentration to decrease COP by approximately 5 mmHg – a meaningful amount when albumin accounts for ~22 mmHg of COP. A publication by Nitta et al. in 1981 supports this number, suggesting that a 1 g/dL drop in albumin concentration correlates to a 5–6 mmHg drop in COP [16].

The type of patient who could benefit most from this tool is the underweight, hypovolemic, or pediatric patient, as these patients are most susceptible to dilution with their proportionally small blood volume relative to the CPB prime. For demonstration, the author would like to present a female, 57 kg patient with a preoperative albumin concentration of 4.4 g/dL. Using the presented tool (or the formulae above) we can calculate that a typical prime volume of 1300 mL requires 47.2 g of albumin in the prime in order to match and maintain this patient’s concentration upon CPB initiation. It is common for an institution to prime with a fixed 12.5 g albumin, and in that instance, the diluted albumin concentration would drop to 3.5 g/dL upon CPB initiation. Another patient, a lean 77 kg male, presents with native albumin concentration of 5.0 g/dL, would require 60.0 g of albumin in at 1200 mL prime to preserve the existing albumin concentration. With a 12.5 g dose of albumin in the prime, the diluted concentration would be 4.3 g/dL. The author would like to underscore the effect that even a seemingly small albumin dilution would create especially as CPB factors discussed above contribute to increased capillary permeability, amplifying the fluid shift created by any change in COP.

Additionally, patients presenting to the operating room with hypoalbuminemia as a result of crystalloid administration without colloid would benefit, and the perfusionist is encouraged to view the trend of albumin concentration prior to pre-operative labs to assess trending.

Conversations about hemodilution usually focus on erythrocyte dilution, however the same dilutional concept occurs with albumin and colloid osmotic pressure. This paper aims to create an awareness and conversation about the practice of albumin and COP maintenance, of which has been limited. A 2001 single-center study published in *Perfusion* reported that 12.5 g of albumin in the CPB prime versus no albumin did not result in a statistically significant difference in postoperative weight gain (*n* = 53), however the difference of 12.5 g albumin has respectively minimal impact on the reported prime volume of 2200 mL [17], respectively. The addition of 12.5 g albumin to this prime would raise the prime concentration from 0.0 g/dL to 0.56 g/dL; a negligible amount, and the authors suggest using a larger volume of albumin to produce a meaningful change in concentration and COP. A revisit of this 2001 study is merited, and the authors aim to compare standard care with model-guided albumin dosing as suggested in this paper in a prospective clinical trial.

With use of the tool created above, perfusionists are enabled to quantify albumin and COP and make more informed decisions regarding fluid management in the cardiac patient. At the very least, this tool and the described formulae provide insight to the perfusionist about the dilution of albumin and the likelihood of sequelae shown to result in cardiac patients that perfusionists are often unaware of, especially when adoption (purchase, validation, training, and maintenance) of a colloid osmometer device may not be feasible. It is the author’s hope that this will inspire further conversation and research with clinical quantification to motivate better, more informed patient care.

## Data Availability

All available data are incorporated into the article.
